# Cost-Effectiveness and Cost-Utility Analysis of the Treatment of Emotional Disorders in Primary Care: PsicAP Clinical Trial. Description of the Sub-study Design

**DOI:** 10.3389/fpsyg.2018.00281

**Published:** 2018-03-06

**Authors:** Paloma Ruiz-Rodríguez, Antonio Cano-Vindel, Roger Muñoz-Navarro, Cristina M. Wood, Leonardo A. Medrano, Luciana Sofía Moretti, PsicAP Research Group

**Affiliations:** ^1^Castilla La Nueva Primary Care Center, Health Service of Madrid, Madrid, Spain; ^2^Department of Basic Psychology II (Cognitive Processes), Faculty of Psychology, Complutense University of Madrid, Madrid, Spain; ^3^Department of Basic Psychology, Faculty of Psychology, University of Valencia, Valencia, Spain; ^4^Department of Psychological Assessment, Universidad Siglo 21, Córdoba, Argentina

**Keywords:** emotional disorders, transdiagnostic cognitive-behavioral therapy, QALYs, cost-utility, primary care

## Abstract

**Introduction:** In the primary care (PC) setting in Spain, the prevalence of emotional disorders (EDs) such as anxiety, depression and somatoform disorder is high. In PC patients, these disorders are not always managed in accordance with the recommendations provided by clinical practice guidelines, resulting in major direct and indirect economic costs and suboptimal treatment outcomes. The aim is to analyze and compare the cost-effectiveness and cost-utility of group-based psychological therapy versus treatment as usual (TAU).

**Methods:** Multicenter, randomized controlled trial involving 300 patients recruited from PC centers in Madrid, Spain, with symptoms or possible diagnosis of anxiety, mood (mild or moderate), or somatoform disorders. Patients will be randomized to one of two groups: an experimental group, which will receive group-based transdiagnostic cognitive-behavioral therapy (TD-CBT); and a control group, which will receive TAU (mainly pharmacological interventions) prescribed by their general practitioner (GP). Clinical assessment will be performed with the Patient Health Questionnaire (PHQ). Direct and indirect costs will be calculated and relevant socio-demographic variables will be registered. The Spanish version of the EuroQol 5D-5L will be administered. Patients will be assessed at baseline, immediately after treatment finalization, and at 6 and 12 months post-treatment.

**Discussion:** To our knowledge, this is the first study to compare TD-CBT to TAU in the PC setting in Spain. This is the first comparative economic evaluation of these two treatment approaches in PC. The strength of the study is that it is a multicenter, randomized, controlled trial of psychotherapy and TAU for EDs in PC.

**Trial registration:** Protocol code: ISCRCTN58437086; 20/05/2013.

EUDRACT: 2013-001955-11.

Protocol Version: 6, 11/01/2014.

## Introduction

Emotional disorders (EDs)—especially anxiety, mood, and somatoform disorders—are the most prevalent types of mental disorders in Spain (Haro et al., 2006). As in many countries, individuals in Spain suffering from EDs typically first consult with their PC GP for diagnosis and treatment. According to a recent study in Spain (Serrano-Blanco et al., 2010), the 12-month prevalence rate for mental disorders among users of the Spanish PC system was 31.2% in Catalonia (Spain). Eighteen point 5% of the patients fulfilled criteria for an anxiety disorder and 13.4% for depression, which are associated with chronic pain, gastrointestinal disorders, and other chronic physical conditions.

Medical treatment usually involves the prescription of psychoactive drugs, most commonly anxiolytics. However, according to clinical practice guidelines, medical therapy is not the treatment of choice; rather, for most EDs, the initial treatment recommendation is psychological therapy. Given this tendency in the Spanish system to prescribe medications, it is not surprising that anxiolytic use in Spain is much higher than in other countries. According to a recent report (OECD, 2015), the median DDD of anxiolytics per 1000 inhabitants in Spain was 52.3 doses—double the median rate (22.3 DDD) reported by the OECD.

The high use of psychoactive drugs in Spain reflects not only the prevalence of EDs, but also underscores the burden of these illnesses. According to Lara et al. (2015), neuropsychiatric disorders were the leading cause of disability for all illnesses in the year 2010, and depressive and anxiety disorders—together with the use of psychopharmacological substances—were the conditions that most contributed to the increase in years lived with disabilities. In view of the above, to accurately assess the costs originated by these disorders, it is essential to consider not only the direct costs of health system utilization, but also the indirect economic burden caused by lost or reduced productivity (Ruiz-Rodríguez et al., 2017).

The total economic cost for mental disorders in Spain is high, but precise figures are not known because published estimates vary widely. According to 2002 administrative data, these costs were estimated to be approximately 1% of the GDP (Oliva-Moreno et al., 2009). By contrast, a more recent estimate of the costs of brain disorders (mental and neurological disorders) in Spain (Parés-Badell et al., 2014), based on rigorous clinical studies on the prevalence and costs of each disease, found that the costs were nearly €84 billion in the year 2010 (approximately 8% of GDP). Remarkably, these costs exceeded the total medical public expenditure, which was €69 billion (6.6% of GDP). According to that study, the costs of mental disorders (excluding neurological disorders) amounted to €46 billion (4.4% of GDP), with depressive, anxiety and somatic disorders accounting for nearly half (€22 billion; 2.2% of GDP) of the total for all mental disorders.

Despite the high prevalence of both anxiety and depressive disorders in Spain, one study found that only 30.5% of PC patients and 31.8% of specialized care patients received “minimally adequate” evidence-based treatment (Fernández et al., 2006). The reason(s) for this lack of adherence to evidence-based clinical practice guidelines is unclear. Consequently, more research is needed to investigate this question so that steps can be taken to improve physician adherence to guidelines and, thereby, improve treatment effectiveness and reduce treatment-related costs, particularly those associated with prescription medications.

Poor adherence to clinical guidelines is not unique to Spain. In the year 2007, a similar problem was identified in the United Kingdom, ultimately leading to the development of a program entitled “IAPT” designed to promote the treatment of EDs in PC with CBT in accordance with the NICE recommendations (NICE, 2011). That initiative has been very successful, resulting in substantially improved treatment outcomes while simultaneously reducing costs (particularly due to decreased drug prescriptions) compared to the usual PC treatment (Radhakrishnan et al., 2013). Indeed, given the success of the IAPT program with EDs, the program has been extended for use in children and in patients with chronic physical conditions (Gyani et al., 2013). Due to the considerable savings of the CBT approach versus drug-based therapies, one recent study estimated that even if the number of patients treated with CBT in the PC setting doubled (from 500,000 to 1 million per year), the net cost would likely be zero if patients with common mental disorders (anxiety and depression) and those with comorbid chronic physical conditions receive CBT (Layard and Clark, 2015).

In Spain, a randomized controlled clinical trial [the Psychology in Primary Care (PsicAP) study] is currently in progress to compare evidence-based psychological techniques [such as transdiagnostic (TD)-CBT] to treatment-as-usual (TAU) (Cano-Vindel et al., 2016) in patients with EDs. Given this context, the present work describes the design of a study being conducted as part of the PsicAP clinical trial (sub-study 2). The aim of this sub-study is to assess and compare the costs associated with psychological and pharmacological treatment approaches for mental disorders in the PC setting in Spain. This study will assess the relationship between cost-effectiveness and cost-utility of these two different treatment approaches. We hypothesize that the costs of switching from TAU to a group-based TD-CBT approach will be justified by the expected additional clinical and social benefits (a superiority trial).

## Methods and Analysis

### Study Design

This a multicenter (see **Appendix 1**, list of PC Centers), randomized controlled trial carried out in Madrid, Spain. Patients will be randomly allocated to one of two groups—an experimental group and a control group—both of which will be treated in a PC setting. The experimental group will receive group-based CBT and the control group will receive TAU (mainly pharmacological interventions). Evaluators will be blinded to the treatment group allocation throughout the evaluation period.

### Patient Recruitment

Patients from the participating PC centers will be recruited by their GP who will provide patients with a document containing detailed information about the study. All patients will be required to sign an informed consent form prior to enrolment. The GP will provide the researchers with the patients’ contact information and relevant data so that the psychologists can arrange a screening test to determine patient eligibility.

The study inclusion criteria for the first phase of recruitment, which will be managed by the GP, are as follows: (1) age range, 18–65 years; (2) previous history of anxiety, depression, or somatoform disorders; (3) WHO ICD-10 criteria for anxiety, depression or somatoform disorders; (4) diagnostic evaluation or clinical suspicion of the presence of at least one of these disorders.

In the second phase of recruitment, clinical psychologists will consider the following inclusion criteria: (1) age, 18–65 years; (2) presence of emotional symptomatology (anxiety, depression, or somatization) with suspected diagnosis of anxiety (generalized or panic attacks), mood (mild and moderate), or somatoform disorders. All candidates will be assessed with the PHQ (Spitzer et al., 1999). The Spanish version validated by Diez-Quevedo et al. (2001) will be used, except for the GAD module, which will be replaced by the GAD-7 validated Spanish version developed by García-Campayo et al. (2012). Patients will be considered eligible for study inclusion if they meet the criteria (based on a decision tree algorithm) for a likely diagnosis of one of these disorders or if the patient has a score ≥ the cut-off score for any of the following PHQ subscales: GAD-7 (generalized anxiety ≥ 10); PHQ-9 (depression ≥ 10); PHQ-13 (somatizations ≥ 5); PHQ-PD (panic attack, ≥ 8 for the first four items of that scale).

Exclusion criteria include the following: diagnosis of a severe mental disorder, such as bipolar or personality disorder (both those described in the patient’s clinical history, treated by specialized mental health services, and those detected through the clinical diagnostic interview, in the second phase of recruitment); presence of a recent, severe suicide attempt; high impairment scores (≥25) on the Sheehan Disability Scale (Sheehan et al., 1996); and legal disability. Additional exclusion criteria include: presence of severe anxiety disorder (e.g., comorbid with substance-use disorders) and/or severe mood disorder (PHQ-9 ≥ 20). Participants who score between 20 and 23 points on the PHQ will undergo a second order assessment conducted by a clinical psychologist to confirm the existence of severe major depression, and any patients with major depression will also be excluded from the trial. Patients will not receive any financial compensation for their participation in the study.

### Sample Size

Two programs were used to determine sample size: (a) G^∗^Power3 (Faul et al., 2007) which prioritizes statistical power, and (b) SSS-CET (Software for Sample Sizes in Cost Effectiveness Trials) which uses a Bayesian approach (Sarker et al., 2013). Based on these analyses, a sample size of 176 participants per group was established (*N* = 352). However, it should be noted that previous investigations have proven that 150 participants are sufficient for a cost-effectiveness study (Rubio-Valera et al., 2013, 2015). Thus, the sample size established for this study is 150 participants per study group (*n* = 300).

#### Assignment of Interventions

##### Allocation

After informed consent is obtained, participants are randomly assigned to either the treatment or control group by a researcher, according to a blind research design, using a computer-generated allocation sequence, thus assuring comparable groups on main outcomes. Each group will include approximately 8–10 patients randomly allocated to the experimental (TD-CBT) or control group (TAU). Subjects will receive the allocation information via email from a graduate student trainee affiliated with the project. The email will also provide login details and website information for the allocated intervention.

##### Blinding

One clinical psychologist will be assigned to the TD-CBT group; importantly, the clinical psychologist involved in the pre- and post-treatment assessment phases will not participate in the TD-CBT therapy. Data managers and statisticians will also be blinded to the treatment allocation.

**FIGURE 1:**
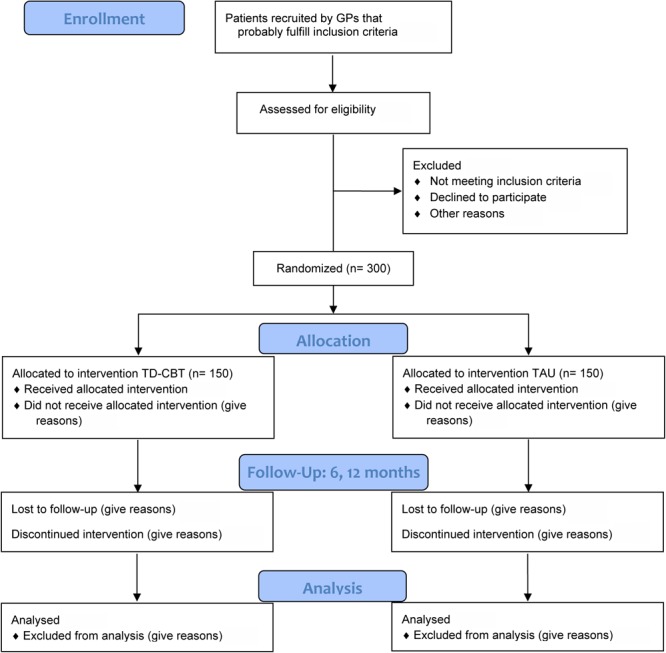
Flowchart of the study.

The flowchart of the study is summarized in **Figure 1**.

### Interventions

#### Experimental Group

The treatment program is included within a collaborative protocol of a stepped-care model of treatment, as recommended by the NICE (2011) guidelines. The first step involves group TD-CBT based on a treatment manual [Cordero-Andrés et al., 2017; González-Blanch et al., 2017; Cano-Vindel (Unpublished)]. The characteristics of this treatment have been described previously by Cano-Vindel (2011). EDs are believed to originate from a dual process of dysfunctional emotional learning over time that involves both cognitive (increasing use of cognitive distortions) and associative (classical conditioning in some significant emotional events) learning. Although this dysfunctional learning tends to lead to symptom chronicity and the development of new, comorbid disorders, the process is considered to be reversible. Moreover, both types of functional relearning (i.e., cognitive and associative) can be achieved with cognitive-behavioral techniques such as cognitive restructuring and behavioral exposure. A transdiagnostic approach (Newby et al., 2015), which involves simplification and economy of resources by applying similar therapeutic procedures to different EDs, will be used in this group intervention. However, the unique characteristics of the cases in each group will be taken into consideration.

Patients in the experimental group will be divided into subgroups of approximately eight patients each and will be treated by clinical psychologists specifically trained for this program. The intervention will include seven sessions (1.5 h/session) delivered over a 24-week period. The contents include the following: psychoeducation, cognitive restructuring, relaxation, behavioral training, and relapse prevention.

#### Control Group Intervention

All patients in the control group will receive standard treatment (TAU) from their GP according to the usual criteria. In most cases, this will involve pharmacological treatment.

### Clinical Evaluation

Patients will be assessed at baseline, immediately after treatment finalization, and at 6 and 12 months post-treatment.

### Instruments

The PHQ is a screening test derived from self-reported tests of the PRIME-MD system (Spitzer et al., 1999), an evaluation system for mental disorders in the PC setting, validated in Spain by Diez-Quevedo et al. (2001). This system contains a number of self-report instruments that can detect the presence of depressive, anxiety, and somatoform disorders (Kroenke et al., 2010).

The PHQ-9 (Kroenke et al., 2001) is an instrument specifically designed to screen for depression; patients are asked to rate each of the 9 DSM-IV criteria. Only symptoms occurring during the 2 weeks prior to the evaluation are considered. Items are rated on a four-point Likert scale from 0 to 3, as follows: 0 (never), 1 (several days), 2 (more than half of the days), and 3 (most days). For a diagnosis of MDD, the algorithm requires a score of 2 or 3 on at least one of the first two symptoms, and must score 2 or 3 on at least 5 of the 9 items (the 9th item-suicide attempts-also counts with a score of 1). A score ≥ 10 is considered the best cut-off point for screening, presenting a sensitivity of 0.88 and specificity of 0.88. McMillan et al. (2010) found that therapeutic success could be assessed with the PHQ-9 as follows: patients with a post-treatment score below the cut-off level (≤9) who experienced a one standard deviation (five point) decrease from pre-treatment scores could be considered in remission, while scores ranging from 10 to 14 indicated minor depression, dysthymia or moderate major depression while scores from 15 to 19 indicated moderately severe major depression, and scores from 20 to 27 indicating severe major depression. Another item (item 10) has been added to evaluate the degree of dysfunction (McMillan et al., 2010). In the Spanish PC setting, PHQ-9 has shown to be a highly satisfactory tool that can be used for screening MDD (Muñoz-Navarro et al., 2017a,c).

The GAD-7 (Spitzer et al., 2006) is used to rate seven common anxiety symptoms presenting during the prior 2 weeks. This instrument can be used not only as a screening test for GAD, but also for other anxiety disorders. The maximum score is 21. Anxiety is classified as mild (≤5 points), moderate (6–14 points), or severe (≥15). The validated Spanish version developed by García-Campayo et al. (2012) was used instead of the original version of the PHQ (Diez-Quevedo et al., 2001). Using a cut-off score of 10 points, the GAD-7 has a sensitivity of 89% and specificity of 82% for GAD. A computerized version of the GAD-7 has been shown to be an excellent screening tool for detecting general anxiety disorder in Spanish PC settings (Muñoz-Navarro et al., 2017b).

The PHQ also contains the DSM-IV symptoms of panic disorder, PHQ-PD (Wittkampf et al., 2011). Patients who answer “yes” to the first four questions and present ≥ 4 symptoms are considered to have a probable diagnosis of panic disorder. Its psychometric properties have been studied with Spanish patients from PC centers (Muñoz-Navarro et al., 2016).

The PHQ-13 includes 13 somatic symptoms, with a maximum score of 26. To detect a probable diagnosis of somatization disorder, at least 3 of the first 13 symptoms must receive the maximum score (two points), and there can be no biological explanation for the somatic disorder. A more recent version—the PHQ-15 (Kroenke et al., 2002), which includes two additional items—has been developed; in the validation study, 88% of the patients who met these criteria had a somatoform disorder; however, psychometric values were lower in later studies (sensitivity, 0.78; specificity, 0.71 (van Ravesteijn et al., 2009; Kroenke et al., 2010).

The Sheehan Disability Rating Scale (Sheehan et al., 1996) is a self-administered test that subjectively evaluates the degree of disability or dysfunction across three life domains—work, social, and family life—using three scales. Two additional items also assess the degree of stress in the last week and the perceived level of social support. The first four items are scored on a 10-point scale ranging from 0 (unimpaired) to 10 (maximum disability). Scores ranging from 1–3, 4–6, and 7–9 indicate, respectively, mild, moderate, or high disability. The fifth item (perceived social support) utilizes the same type of scale, but this is expressed as a percentage, ranging from 0% (non-existent support) to 100% (perfect support).

### Economic Assessment

In this study, our aim was to compare the association between cost-effectiveness and cost-utility of the two treatments. The economic evaluation will be conducted by recording variables (see below) obtained from patient medical records and patient interviews.

To quantify direct costs, the following variables (only related to EDs) will be registered and assessed: number of patient consultations with psychologists and/or psychiatrists; number of patient visits to the PC centers (GP, nurse, or social worker); number of patient visits to public or private hospitals; number of visits to clinics/physicians associated with private health insurance companies; number of road traffic, workplace, or domestic accidents; number of hospitalizations and emergency room visits; medications used (name of drug, daily dose, and duration of treatment); consultations with other therapists (i.e., podiatrists, physiotherapists, and dieticians). Diagnostic tests requested by the GP and/or specialists to assess physical symptoms secondary to the emotional disorder(s) will be included. Indirect costs will include all days off from work. If a replacement worker is needed, then this will be registered as an indirect cost as well.

All the variables will be collected for the following time points: 3 months before study inclusion; at baseline; immediate post-treatment; and at 6 and 12 months post-treatment.

After providing written informed consent, the participants will be registered in the treating center. Pre- and post-treatment assessments are to be carried out using computerized self-reported screening tests. All pretreatment assessments will be performed at the treating PC center after scheduling an appointment with the clinical psychologist. A computer with Internet access is used to collect data. All data are stored on a general virtual website (surveymonkey.com). At all post-treatment follow-up assessments, the same instruments will be completed in person at the treating center.

#### Retention

If necessary, we will send the participant a link by email to enable the patient to complete the computerized measures at home. Patients are contacted by phone to encourage completion of the questionnaires. Patients who discontinue or drop out of treatment will still be invited to complete the post-treatment follow-up assessments, particularly the first post-treatment assessment.

### Main Outcomes

The main outcomes are the clinical symptoms assessed by the PHQ: depression, anxiety, panic, and somatic symptoms, as well as economical variables and EuroQol 5D outcomes in terms of QALYs.

### Secondary Outcomes

Measures of disability will be included in the secondary outcomes. The following socio-demographic variables will be registered and analyzed: sex; marital status (married, divorced, widow/widower, separated, single, cohabitation); age; education level (none, elementary education, secondary education, university education, post-graduate degree); work status (part-time employee, full-time employee, temporary incapacity for work or permanently disabled, unemployed); and household income level (<€12,000; €12,001 - €24,000; €24,001 - €36,000; >€36,000).

### Statistical Analysis

#### Analysis of Clinical Effectiveness

This study will be conducted on an intention-to-treat basis. Both groups will be compared to verify that there are no significant between-group differences at baseline. For comparisons, the ANOVA method will be used for continuous variables and the Chi-square test for categorical variables. Subsequently, a repeated measurement of ANOVA, including all variables over time, will be performed to assess whether missing values respond to a random or non-random pattern. If the missing values are greater than 5% the Expectation Maximization (EM) will be used as a data imputation method, as recommended by the literature (Schlomer et al., 2010).

#### Description of Cost Analysis

Direct costs of health care will be estimated by adding the costs derived from medication use (antidepressants, hypnotics, sedatives, and anxiolytics), medical tests, use of health-related services, and staffing costs. The cost of medications will be calculated by determining the price per milligram (mg) during the study period according to the Vademecum International (Vidal, 2014), including value-added tax. Total costs of medications will be calculated by multiplying the price per mg by the daily dose (mg) and the number of days of drug treatment.

The main source of unit cost data related to public medical tests and use of health services will be obtained from the fee information published by the Official Government Journal of the Autonomous Community of Madrid (2013). Indirect costs will be calculated according to the number of days on sick leave multiplied by the minimum daily wage in Spain for 2015 (€21.62). Finally, total costs will be calculated by summing the total direct and indirect costs. The unit costs will be expressed in Euros (€) based on 2015 prices.

Currently, there is no reference fee for the TD-CBT intervention that will be used with the experimental group. The estimated cost of a consultation with a public sector GP in Spain is €39 (not including complementary tests). Given that salaries for psychologists in Spain are similar to those of GPs, we will use this amount (€39) to calculate the cost of the TD-CBT intervention (i.e., €39 multiplied by the number of assessment and treatment sessions, €39 × 9 = €351). Since the GP consultation fees are understood to include the cost of office space, we will not add any additional costs for office rent to the cost of the therapy. Accordingly, the total cost of the evaluation and psychological intervention (nine sessions) per person is estimated at €43.87 (€351/8).

#### Cost-Effectiveness Analysis

The cost-effectiveness analysis will be carried out by calculating the incremental cost-effectiveness ratios (ICER), defined as the difference in mean costs divided by the increase in effectiveness of the various therapeutic alternatives.

#### Cost-Utility Analysis

Cost-utility analysis is a method of economic evaluation based on preferences or utilities rated by individuals; in other words, the value that an individual assigns to their health status. Thus, the economic evaluation depends on the social perspective of the individual, in which patients express preferences about their health. The EuroQoL-5D Questionnaire (EQ-5D – Spanish version) will be used for the cost-utility analysis. This is a generic QOL instrument consisting of five domains, as follows: mobility; anxiety/depression; pain/discomfort; daily activities; and self-care. These five domains are coded into five severity levels, as follows: no problems; slight problems; moderate problems; severe problems; extreme problems. In this way, 3125 distinct health states can be established. Health states enable us to obtain a population-based preference score per country (the EQ-5D index). The assigned values range from 0 (death) to 1 (perfect health). In addition, this index includes a VAS similar to a thermometer, which measures health from 0 (worst imaginable health state) to 100 (best imaginable health state) (Rabin et al., 2015).

Through the EQ-5D health questionnaire, we will calculate utility as QALYs using the Spanish fees described above.

Using these data, we will calculate the incremental cost-utility ratios, defined as the difference in mean costs divided by the difference in mean QALYS. Given that the duration of this study is only 12 months, neither costs nor outcomes are subject to discount. To obtain more precise cost-utility ratios, these ratios will be calculated by means of the bootstrapping method (a resampling method), which involves creating a large number of samples with replacement from the original sample data, thus allowing for a distribution that better resembles the actual distribution of the population from which the original data was drawn. In this way, the study will generate 1000 random samples which will be used to define the confidence intervals for the cost-utility ratios.

Missing-data analysis will be computed using Student’s *t*-test and chi-square tests. Variables included in the analysis will be severity level, gender, and age; this will allow us to ascertain whether unexpected missing data due to participant dropout are related to chance or not.

#### Sensitivity Analysis

A sensitivity analysis will be performed to test the robustness of the cost-effectiveness and cost-utility results.

## Discussion

In Spain, the entry point to the health care system is the PC center, and the GP is the first point of contact with the health care system for most patients in Spain with an ED. According to the NICE clinical practice guidelines (NICE, 2011), psychological therapy (i.e., CBT) is the initial treatment of choice for these disorders. However, in Spain, no studies have yet evaluated a group TD-CBT approach in PC centers. In the PsicAP clinical trial, our aim is to demonstrate that the TD-CBT treatment approach is both feasible and effective.

Before a new health care program can be widely implemented in any health care system, it is necessary to not only demonstrate the effectiveness of the program, but also to assess the intervention in terms of costs and benefits. An economic assessment is crucial given that, in most countries, resources are limited while demand is potentially unlimited. In Spain, economic evaluations of health care intervention programs in PC are rare; in the treatment of EDs, such evaluations are practically non-existent, despite the high prevalence (Haro et al., 2006) and high costs to society (Parés-Badell et al., 2014) of these disorders. For all these reasons, it is essential to conduct an economic sub-study as part of the PsicAP clinical trial.

In the present article, we have described the methodology that will be used to assess the relationship between cost-effectiveness and cost-utility. The strength of the study is that it is a multicenter, randomized clinical trial. Moreover, two different types of economic evaluation will be carried out. The analysis will help us establish a relationship between treatment cost and effectiveness based on the health care outcomes measured through previously validated questionnaires. In addition, this analysis will also help to demonstrate the relationship between cost and utility based on the QOLdata (measured in terms of number of QALYs gained), an internationally recognized measurement that will allow us to compare our findings to other studies.

A limitation of this study is that health care in Spain is managed independently in each of the 17 autonomous regions of Spain. To reduce variability in this sub-study, we will use the community of Madrid as our reference area for the cost analysis. However, the data will be obtained from numerous PC centers in this region to obtain a more representative sample.

If our hypothesis—that the costs of switching from TAU to a group TD-CBT approach is justified by the clinical and social benefits—is confirmed, this could have a profound impact on the treatment of EDs in the PC setting in Spain. Considering that EDs affect nearly one-third of PC patients, this would have important implications, as evidence-based interventions recommended by clinical practice guidelines should lead to better outcomes and improved QOL in our patients. Moreover, if our expected results are achieved, it would be possible to significantly reduce the cost of treating these disorders—currently, 2.2% of GDP in our country—by replacing pharmacotherapy with group-based CBT interventions in this patient population.

### Ethics and Dissemination

Patients will be required to give their informed consent before enrolment in the study. In addition, patients will be randomly allocated to the treatment or control group and will not know which treatment they will receive until allocation. The information sheet will clearly explain that participation is completely voluntary and that participants can withdraw from the study at any time without any negative consequences.

The proposed study respects and will abide by the existing legislation and other laws related to the project, in the field of ethics, animal experimentation, and biosecurity. This study will be conducted in accordance with the Declaration of Helsinki, the Council of Europe Convention concerning human rights and biomedicine, the UNESCO Universal Declaration on the Human Genome and Human Rights, and the Convention for the Protection of Human Rights and Dignity of the Human Being with regard to the Application of Biology and Medicine (Oviedo Convention on Human Rights and Biomedicine).

The study protocol was approved by the Clinical Research Ethics Committee of the Valencia Primary Care Organization (CEIC-APCV), the clinical ethics research committees of the participating centers and by the Spanish Agency of Medicines and Medical Devices (AEMPS).

## Ethics Statement

### Research Ethics Approval

This is a sub-study into a multi-center Randomized Clinical Trial with medication (N EUDRACT: 2013-001955-11 and Protocol Code: ISRCTN58437086) promoted by the *Psicofundación* (Spanish Foundation for the Promotion, Scientific and Professional Development of Psychology) and approved by the Corporate Clinical Research Ethics Committee of Primary Care of Valencia (CEIC- APCV) (as the national research ethics committee coordinator) and the Spanish Agency of Medicines and Health Products. Approval was received by both agencies in November 2013, prior to study initiation in December 2013.

The CEIC-APCV approved the trial in three centers in the autonomous communities of Valencia (1), Balearic Islands (1), and Castilla la Mancha (1). The study was also approved by the three first local Ethics Committees: the CEIC-APCV, the Clinical Research Ethics Committee of the Hospital Universitario de Albacete (CEIC-HUA), and the Clinical Ethics Committee of the Balearic Islands (CEI-IB).

### Protocol Amendments

Six protocol amendments have been presented during the course of this trial:

*Amendment 1:* One PC center was added to the autonomous communities of the Basque Country and was approved by the Clinical Research Ethics Committee of Euskadi (CEIC-E). Also, a sub-study (sub-study 1) was presented to conduct the study of the psychometric properties of the PHQ subscales of the PHQ-9, PHQ-PD, and GAD-7 with 15% of the larger sample. This sub-study has been conducted in four PC centers located in the autonomous communities of Valencia (1 center), Balearic Islands (1), Basque Country (1) and Castilla la Mancha (1). The sub-study was also approved by the three first local Ethics Committees: the CEIC-APCV, the Clinical Research Ethics Committee of the Hospital Universitario de Albacete (CEIC-HUA), the Clinical Research Ethics Committee of Euskadi (CEIC-E), and the Clinical Ethics Committee of the Balearic Islands (CEI-IB).

*Amendment 2:* Nine centers located in the Community of Madrid were added to the study. The Clinical Research Ethics Committee of Madrid approved this amendment, as did the national Ethics Committee, the CEIC-APCV.

*Amendment 3:* One PC center was added to the group of centers in the autonomous community of Valencia. This center thus becomes a full participant in the trial and the sub-study (bring the number of PC centers in sub-study 1–5). Also, several changes to the first version of the protocol were made, including the use of the SCID-I to confirm severe MMD and questions to confirm high disability on the SDD, as described above. Also, new researchers were added to the study. The amendment was approved by the national Ethic Committees (the CEIC-APCV) and by the relevant local ethics committees.

*Amendment 4:* Three PC centers, two in Andalusia (2) and one in Cantabria (1), were added to the list of participating centers. Also, sub-study 2—a study of the cost-efficiency measures that are conducted in the PC centers in Madrid and Valencia—was presented. Several changes to the next version of the protocol were made, including the telephone follow up post-treatment (see “*Therapist Training”* section above). Finally, new researchers were added to the study. The amendment was approved by local Ethic Committees, the Clinical Research Ethics Committee of Córdoba (CEI-C), and the Clinical Ethics Committee of the Cantabria (CEIC) and national Ethic Committee, the CEIC-APCV.

*Amendment 5:* Five PC centers were added to the autonomous communities of Madrid (2) and Valencia (3) to conduct the trial. Also, new researchers were added to the study. The amendment was approved by local Ethic Committees, the Clinical Research Ethics Committee of Madrid (CEIC-M), and the local and national Ethic Committee, the CEIC-APCV.

*Amendment 6:* Six PC centers were added to the autonomous communities of Cataluña (2), Galicia (2) and in Navarra (2) to conduct the trial. Also, new researchers were added to the study. The national legislative norms have been modified in Spain, and now only one national Ethics Committee is required for RCTs. As a result, this amendment was approved by the national Ethics Committee, the CEIC-APCV. Two new sub-studies were also presented. Sub-study 3 is a modification of the protocol design (stepped wedge trial design), which will be conducted in 2 PC centers in Barcelona (Catalonia). Sub-study 4 is a study to assess the value of using the PHQ-4 to detect EDs in PC centers before the patient sees the GP; the aim is to reduce misdiagnoses of EDs and to accelerate referral to the clinical psychologist in the second phase of the recruitment process. This will allow us to determine if the ultra-short measure of the PHQ-4 is an appropriate tool to help GPs to detect EDs and to reduce the large number of false negatives. If results are as expected, this may lead to a proposal for a new referral model in Spanish PC centers.

### Consent

#### Patient Informed Consent

Prior to study participation, all patients receive written and oral information in the *patient information sheet* about the content and extent of the planned study. This includes information about the potential benefits and risks for their health. Patients who agree to participate are required to sign the informed consent form. In the case of patients who withdraw from the study, all data will be destroyed or the patient will be asked if he/she agrees to allow the use of existing data for analysis in the study.

Patient participation in the study is completely voluntary and participants can withdraw at any time with no need to provide reasons and without negative consequences for their future medical care. The protocols used in this study pose no risk whatsoever to the participants. CBT is non-invasive at the cognitive level, except with regards to learning or teaching.

### Confidentiality

The study is conducted in accordance with the Spanish Data Security Law. All professionals participating in the study agreed to adhere to the Helsinki Declaration and to Spanish law. All health care professionals participating in the study are required to sign a form indicating their agreement to adhere to the above-mentioned declaration and Spanish law.

The patient names and all other confidential information fall under medical confidentiality rules and are treated according to Spanish Data Security Law. The patient questionnaires are collected by nurses and mailed by secure transport to the study center in Madrid. All study-related data and documents are stored on a protected central server and saved in an encrypted database.

The project complies with current guidelines in Spain and EU for patient protection in clinical trials with regards to the collection, storage and the keeping of personal data. Only direct members of the internal study team can access the data.

## Availability of Data

The study data are only available upon request. The name(s) of the contact person(s) to request data are available upon request to all interested researchers. Legal and ethical restrictions make data available upon request and are in accordance with the nature of the data collection.

This is a multi-center Randomized Clinical Trial with medication (No. EUDRACT: 2013-001955-11 and Protocol Code: ISRCTN58437086) promoted by the *Psicofundación* and approved by the Corporate Clinical Research Ethics Committee of Primary Care of Valencia (CEIC- APCV) (as the national research ethics committee coordinator) and the Spanish Medicines and Health Products Agency. The CEIC-APCV have some availability restrictions, as a part of the legal and ethical control data of a Randomized Clinical Trial with medication.

Data are available from the promoter (Spain) for researchers who meet the criteria for access to confidential data. Contact: *Psicofundación* (Spanish Foundation for the Promotion, Scientific and Professional Development of Psychology). Address: Calle Conde de Peñalver, 45, 5o izquierda, 28006 Madrid, Spain.

## Members of the PsicAP Research Group

M^a^. Dolores Gómez Castillo, Patricia Tomás Tomás, Gonzalo Jiménez Cabré, Juan Antonio Moriana, Antonio Capafons Bonet, María Rosa Pizà, Ignasi Ramírez-Manent, Laura Agüero, Mónica Rodríguez-Enríquez, Estefanía Salgado Kvedaras, Jorge Perpiñá González, Florian Schmitz, Carmen Abellán Maeso, Ana M^a^. Agudo Rodrigo, M^a^. del Mar Aguilar Uceda, Manuel Aires López, Ana María Alayeto Sánchez, Dolores Alfonso Doménech, Vicenta Almonacid Guinot, Luciana Moretti, Sinuhe Alvarado Torres, Sonia Álvarez Gómez, Luisa M^a^. Andrés Arreaza, Ana María Arnaiz Kompanietz, M^a^. Pilar Arranz García, Eugenia Avelino Hidalgo, M^a^. José Ávila Sánchez, Josune Barbero Goicoetxea, Manuel Barragán Solís, Pilar Barroso López, Sofía Bauer Izquierdo, Carmen Benavente Torres, Pilar Bermejo Ortega, Greta Borrás Moreno, Alejandro Buendía Romero, Carlos Buiza Aguado, José Caballero Moral, Nieves Caparrós Ezpeleta, Alejandro Casado Martínez, Cristina Casado Rodríguez, Consuelo Castiblanque Ballesteros, Francisca Ceinós Vicente, Patricia Cordero Andrés, Ana Costa Alcaraz, Isabel de Andrés Cara, Victoria de la Riva Casares, Bárbara Díaz Gómez, M^a^. Dolores Domínguez Manrique, Soledad Escolar Llamazares, Encarnación Espinosa de los Monteros Zayas, Aurora Fabero Jiménez, M^a^. Antonia Font Payeras, M^a^. Rosa Fraile Gómez, Mariona Fuster Forteza, Montserrat Gallart Aliu, Olvido García Jaén, Carmen García Palacios, Mar García-Moreno, Yolanda Garnica Cascales, Miguel Gárriz Vera, M^a^. Concepción Gómez Martín-Sonseca, M^a^. Francisca Gómez Rodríguez, César González-Blanch Bosch, Ana M^a^. Hermosilla Pasamar, Fernando Hernández de Hita, Margarita Herrero Delgado, Josefa Jaimez Moreno, Amale Jáuregui Larrabeiti, Juan Agustín Jiménez Luque, Antonio Jiménez Moreno, Antonio León Dugo, Carlos Lillo De la Quintana, Joaquín T. Limonero García, María Lleras de Frutos, Anna Llorca Mestre, Francisco López Ortiz, Lourdes Luceño Moreno, Pilar Madrid Almoguera, Sonia Martín de la Sierra Fuentes, Rebeca Martínez Bustos, Ainoa Mateu Mullor, Mercedes Matilla Caballero, Rafael Medina Reyes, Itsaso Mendizábal Gallestegui, Susana Merino Martín, Gonzalo David Moneva Vicente, M^a^. Isabel Montejo Villa, Antonio Montero Cantero, Julio Montoya Fernández, Rosario Morales Moreno, Laura Morante Hernández, Eliana M^a^. Moreno Osella, Luciana Moretti, Francisco Javier Muñoz Mora, Rafael Muñoz Sánchez-Villacañas, Juan Elías Murillo Céspedes, Soledad Nevado Roldán, Rosario Ortíz Fernández, Elia Peiró Martí, María Olga Peña Peña, Montserrat Pérez Fernández, Juana Pérez Girón, Nathalie Pérez Lizeretti, Aranzazu Pérez Medina, Pilar Pérez Ortín, José Jorge Pérez Pascual, Bartolomé Pérez Pérez, María Pineda Alonso, Almudena Pinilla Carrasco, Jorge Juan Prada Pérez, Marta Quintanilla Santamaría, Ana Isabel Quiñones Gómez, María Teresa Recio García, Marcelino Requena Gallego, Mercedes Ricote Belinchón, Yolanda Rincón Villareal, Geoffrey Ritho Luhunga, M^a^. Ángeles Rivas Marra, Mariano Robres Oliete, Virginia Rodríguez Coronado, Mercedes Rojo Tardón, Ana M^a^. Roldán Villalobos, M^a^. Teresa Rubio Rubio, M^a^. Jesús Ruiz Hernández, Jesús Ruiz López, Lorenza Ruz Torres, Manuel Salcedo Espinosa, Monika Salgueiro, María Luisa Sánchez Benitez de Soto, Emilio Sánchez Caballero, María Teresa Sánchez Villares Rodríguez, Isabel Sepúlveda Gómez, María Serrano Miralles, Victor Julián Suberviola Collados, Beatriz Talavera Velasco, Javier Torres Ailhaud, Olga Umaran Algageme, Alazne Unanue Ortega, Iñigo Valdivielso Moneo, Cristina Valle García, Antonio Varo Soriano, José Fernando Venceslá Martínez, M^a^. Jesús Villa Pérez, Esperanza Villar Coloma, and Laura Yuste Hidalgo.

## Author Contributions

PR-R wrote the first draft of the manuscript and revised it and managed the literature searches of the manuscript. AC-V designed the study and wrote the protocol. RM-N assisted with the preparation and proof-reading of the manuscript and obtained the ethical approval. CW and LAM assisted with the preparation and proof-reading of the manuscript. LSM assisted with the preparation of the manuscript. PsicAP Research Group is a large equipment of researchers and professionals who are doing possible the recruitment and other tasks into the PsicAP trial.

## Conflict of Interest Statement

The authors declare that the research was conducted in the absence of any commercial or financial relationships that could be construed as a potential conflict of interest.

## APPENDIX

### Appendix 1 | List of PC Centers

#### Madrid:

Centros de Salud de “Mar Báltico”, “Ciudad de los Periodistas”, “Arroyo de la Vega”, “Castilla La Nueva” (Fuenlabrada), “Pacífico”, “Reyes Magos” (Alcalá de Henares), “Aranjuez”, “Legazpi”, “Juncal” (Torrejón), “Aquitania” y “Panaderas” (Fuenlabrada).

## Abbreviations

AEMPS: Agency of Medicines and Medical Devices
ANOVA: analysis of variance
CBT: cognitive behavioral treatment
CEIC-APCV: Clinical Research Ethic Committee of the Valencia Primary Care Organization
DDD: defined daily dose
DSM-IV: Diagnostic and Statistical Manual of Mental Disorders
€: Euros
EDs: emotional disorders
EQ-5D: EuroQol five dimensions questionnaire
GAD-7: Generalized Anxiety Disorder-7
GAD: generalized anxiety disorder
GDP: gross domestic product
GP: general practitioners
IAPT: Improving Access to Psychological Therapies
MDD: major depressive disorder
NICE: National Institute of Clinical Excellence
OECD: Organization for Economic Cooperation and Development
PC: primary care
PHQ-9: Patient Health Questionnaire-9 Module of Major Depression
PHQ-13: Patient Health Questionnaire Somatic Symptoms, Spanish version
PHQ-15: Patient Health Questionnaire Somatic Symptoms
PHQ-PD: Patient Health Questionnaire Module for Panic Disorder
PHQ: Patient Health Questionnaire
PRIME-MD: Primary Care Evaluation of Mental Disorders
QALY: quality-adjusted life years
QOL: quality of life
TAU: treatment as usual
TD-CBT: transdiagnostic cognitive behavioral therapy
VAS: visual analog scale
WHO: World Health Organization
